# ‘The long tail of Covid-19’ - The detection of a prolonged inflammatory response after a SARS-CoV-2 infection in asymptomatic and mildly affected patients

**DOI:** 10.12688/f1000research.27287.2

**Published:** 2021-01-08

**Authors:** Ivan Doykov, Jenny Hällqvist, Kimberly C. Gilmour, Louis Grandjean, Kevin Mills, Wendy E. Heywood

**Affiliations:** 1 Translational Mass Spectrometry Research Group, University College London Institute of Child Health, London, UK; 2 Clinical and Movement Neurosciences, University College London Queen Square Institute of Neurology, London, UK; 3 Great Ormond Street Children's Hospital NHS Foundation Trust, Great Ormond Street, London, WC1N 3JH, UK

**Keywords:** Sars-CoV-2, mass spectrometry, inflammation, biomarker, proteomics

## Abstract

‘Long Covid’, or medical complications associated with post SARS-CoV-2 infection, is a significant post-viral complication that is being more and more commonly reported in patients. Therefore, there is an increasing need to understand the disease mechanisms, identify drug targets and inflammatory processes associated with a SARS-CoV-2 infection. To address this need, we created a targeted mass spectrometry based multiplexed panel of 96 immune response associated proteins. We applied the multiplex assay to a cohort of serum samples from asymptomatic and moderately affected patients. All patients had tested positive for a SARS-CoV-2 infection by PCR and were determined to be subsequently positive for antibodies. Even 40-60 days post-viral infection, we observed a significant remaining inflammatory response in all patients. Proteins that were still affected were associated with the anti-inflammatory response and mitochondrial stress. This indicates that biochemical and inflammatory pathways within the body can remain perturbed long after SARS-CoV-2 infections have subsided even in asymptomatic and moderately affected patients.

## Introduction

As more and more people are recovering from SARS-CoV-2 infection, one of the growing concerns is the increasing reports of the post viral fatigue symptoms or ‘long Covid’. This phenomenon is defined as not recovering for several weeks or months following the start of symptoms and whereby patients present with chronic and recurrent fatigue for weeks and even many months after a SARS-CoV-2 infection ^1, 2^. Understanding the effects and complications of ‘long Covid’, and then managing it, is the next challenge for public health services. Currently the UK is increasing its testing capacity for virus detection and antibody detection, but there still remains a gap in the understanding and diagnosis of long Covid.

Work has been performed to characterise the inflammatory response to SARS-CoV-2 infection in relation to disease severity ^3^. There has been controversy as to whether severity is associated with a hyperinflammatory cytokine storm or failure of host protective immunity that results in unrestrained viral dissemination and organ injury. What has made addressing this question challenging has been the lack of diagnostic tools to evaluate immune function in Covid-19 infections. There are sets of simple but expensive immunoassay panels that are commercially available to look at known key inflammatory proteins such as cytokine panels; however, these only give information on known pathways and limit discovery of novel or less defined inflammatory responses. Targeted proteomics using mass spectrometry can also quantitate multiple diagnostic proteins without use of antibodies. Proteins can be easily added or removed from a panel thereby providing a custom tailored approach. This is ideal in addressing the need for evaluating less understood or defined immune response pathways. Novel assays for virus detection have been already developed using targeted mass spectrometry ^4, 5^ but there are no assays available yet to look at the symptoms for the diagnosis or understanding of ‘long Covid’.

**Figure 1.**
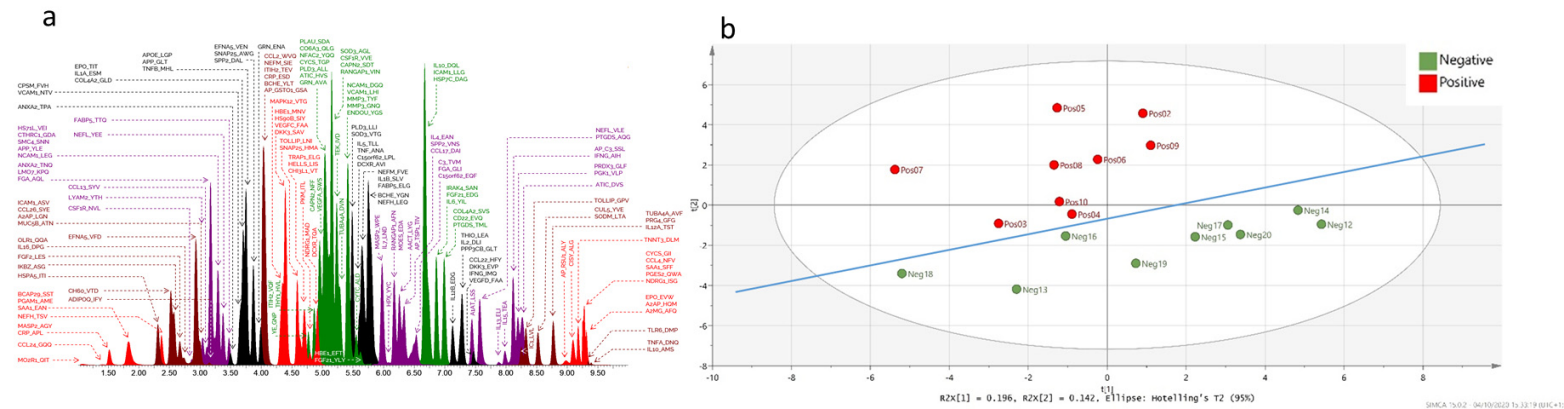
Multivariate analysis of post infection serum inflammation profile. **a**) Representative overlaid chromatogram of the multiplex inflammation panel. Protein identifiers indicated by name and followed by first three amino acids of the peptide. **b**) Principle component analysis score plot of 10 SARS-Cov-2 infected patients >40 days post infection and 10 negative controls.

From a previous study (unpublished reports) we developed a custom targeted mass spectrometry based assay panel that looks at up to 96 pro- and anti-inflammatory associated proteins ( Figure 1a; see Table 1 on protocols.io ^6^). Some of the pathways relevant to the proteins included in the multiplex include upstream regulation of cytokine and glucocorticoid expression; calpain activation; aging associated T-cell production and heat shock protein mediated immunostimulatory ‘danger signals’ for the innate and adaptive immune systems. Our hypothesis was long Covid symptoms could be related to a lingering ‘tail’ and an abnormal inflammatory response to an infection, by a type of virus the body has not seen before. We applied this assay to a cohort of samples taken from healthcare workers who had tested positive for SARS-CoV-2 infection by PCR and were either asymptomatic or had only a mild infection. Samples were taken at least 40–45 days post infection and demonstrated a positive antibody test. We compared these with serum from healthcare workers with a negative antibody test, no reported infection and no positive PCR test.

## Methods

### Ethical statement

Samples were identified from the Health Research Authority approved project Co-Stars (Great Ormond Street Hospital NHS Trust COSTARS, IRAS 282713, ClinicalTrials.gov Identifier: NCT04380896, registered May 8 ^th^ 2020) and all participants provided informed written consent.

### Samples

A pilot group of 10 positive and 10 negative samples covering a broad age range was selected as proof of principle for this assay. The negative group was 60% female with an age-range of 21–57, median 38 years. The positive group was 69% female, with an age range 31–66 and median age of 44 years. Of the positive patients, seven were asymptomatic and six had loss of taste/smell or had abnormal taste/smell. None were admitted to hospital or reported other symptoms.

### Multiplex assay

The detailed method for the multiplex assay is published and available at protocols.io ^6^. Briefly, yeast enolase internal standard was added to serum samples. Proteins were precipitated and trypsin digested to peptides. Peptides were desalted, separated by reverse phase chromatography over a 16 min acetonitrile gradient and analysed on a Waters Aquity UPLC system coupled to a Xevo TQ-S mass spectrometer.

### Analysis

Raw data was acquired using MassLynx v 4.1 in multiple reaction monitoring mode. Raw files were processed using Skyline v 19. Protein-Peptide sequences were obtained from www.uniprot.org and settings optimised using custom synthesised peptides (Genscript USA). Peak intensity data were normalised to a spiked internal standard protein yeast enolase (Sigma, UK). Normalised data were exported to Microsoft Excel and analysed using SIMCA v 15 (Umetrics, Sweden) for multivariate analysis and Graphpad prism v 6 was used for statistical analysis.

## Results

**Figure 2.**
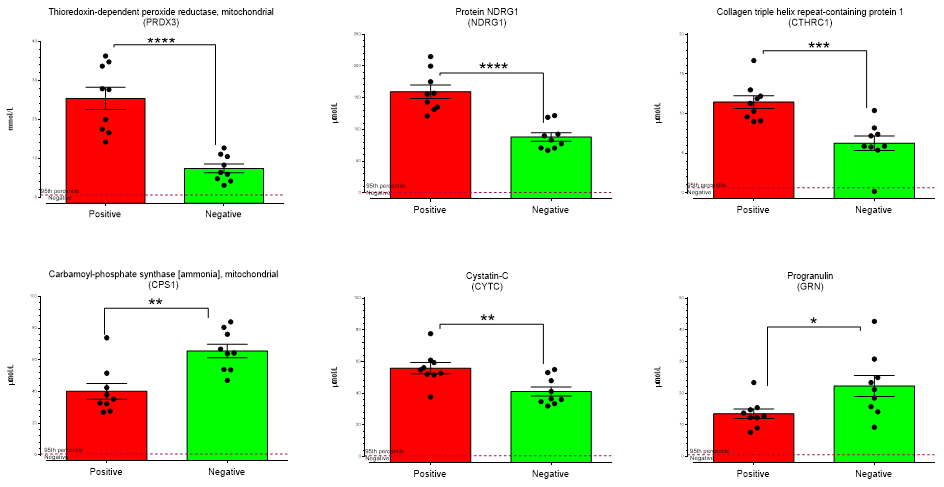
Altered proteins in post infection serum. Proteins significantly affected (p< 0.001) by non-parametric statistical analysis in the serum of >40 day post SARS-Cov-2 infected healthcare workers **** p<0.0001, ***p<0.001, **p<0.01, *p<0.01.

A representative overlaid chromatogram of the proteins included in the multiplex assay is show in Figure 1a. Multivariate analysis of all inflammatory proteins measured in the control and SARS-CoV-2 positive patients is shown in Figure1b. The score plot that shows the first two principal components indicates a clear separation of the positive and negative samples indicating the serum immune profile from people infected with SARS-CoV-2 is still significantly affected even 40 days post-infection. Univariate analysis revealed six proteins ( Figure 2) from the entire multiplex panel were significantly altered. The majority of these proteins are either anti-inflammatory or associated with the stress response. Two proteins originate from the mitochondria, peroxiredoxin 3 (PRDX3) and carbamoyl phosphate synthase (CPS1). PRDX3 is a known antioxidant. Its increase in serum of patients infected with SARS-CoV-2 is likely indicative of continued mitochondrial stress response. CPS1 is a major mitochondrial urea cycle enzyme in hepatocytes. Serum CPS1 originates from the bile duct and is usually rapidly cleared by peripheral blood mononuclear cells ^7^. It is possible that basal levels of CPS1 in serum are reduced in patients infected by SARS-CoV-2 due to increased circulation and activity of peripheral blood mononuclear cells.

N-Myc downstream regulated gene 1 (NDRG1) is a cytosolic protein with many biological functions ^8^. Its role in the immune response is undefined but deficiency of NDRG1 affects the differentiation process of macrophages ^9^ and maturation of mast cells ^10^. Collagen triple helix repeat containing 1 (CTHRC1) is anti-inflammatory and promotes wound healing by recruiting M2 macrophages and regulating the TGF-β and Notch pathways ^11^. This increase of CTHRC1 indicates tissue damage has occurred even in moderately affected patients.

Cystatin C is a protease inhibitor and extracellular levels are used as a biomarker for disease prognosis in cancer, cardiovascular disease, and inflammatory lung disorders ^12^. In mice serum cystatin C is controlled by the anti-inflammatory cytokine IL10 of which increasing levels suppress cystatin C expression ^12^. A longitudinal study looking at immune mediators show IL10 levels are significantly elevated in only severe cases of SARS-CoV-2 infection at four weeks post infection and are not affected at four weeks in mild cases ^13^. This would corroborate with what we observe for cystatin C as the mild patients have increased cystatin C that is not being suppressed by higher IL10 levels. We also observe a slight reduction in serum progranulin. Progranulin plays a fundamental role in the immune response which is better defined within its role in neurodegenerative disorders ^14^ but the relevance of serum progranulin is not fully understood. It appears to have a pro-inflammatory role in adipocytes in diabetes ^15^ and an anti-inflammatory protective role in the vascular endothelium against inflammatory reactions ^16^.

## Conclusions

Remarkably, even in patients who have suffered from an asymptomatic or mild SARS-CoV-2 infection, after 40 days post-infection they still exhibit a significantly raised group of biomarkers involved in inflammation and the stress response. This initial data using a custom designed inflammatory marker panel applied to mildly affected patients identifies potential drug targets, provides insight into the post infection inflammatory response. This approach using targeted proteomic technology has potential for application on further well-defined sample cohorts to understand what is abnormal about post infection inflammatory response in ‘long covid’ patients.

## Data availability

### Underlying data

ProteomeXchange: Underlying mass spectrometry data on ProteomeXchange. Accession number PXD022159.

Underlying mass spectrometry data is also available on PanoramaWeb at https://panoramaweb.org/x1eZmn.url.
