# Supplementary material for: ‘The long tail of Covid-19’ - The detection of a prolonged inflammatory response after a SARS-CoV-2 infection in asymptomatic and mildly affected patients
Source: F1000Res. 2021 Jan 8;9:1349. Originally published 2020 Nov 19. [Version 2] doi: 10.12688/f1000research.27287.2 (PMC7745182; doi:10.12688/f1000research.27287.2)
Supplement: Peer review history

# Peer review: Reviewer response for version 2

**DOI:** 10.5256/f1000research.32778.r76956

**Published:** 2021-01-15

**Authors:** Paul Skipp

**Affiliations:** 1 Centre for Proteomics Research, University of Southampton, Southampton, UK

No further comments.

Is the work clearly and accurately presented and does it cite the current literature?

Partly

If applicable, is the statistical analysis and its interpretation appropriate?

Yes

Are all the source data underlying the results available to ensure full reproducibility?

Yes

Is the study design appropriate and is the work technically sound?

Yes

Are the conclusions drawn adequately supported by the results?

Yes

Are sufficient details of methods and analysis provided to allow replication by others?

Yes

Reviewer Expertise:

Precision Medicine, Proteomics, Clinical Proteomics, Data Science, Biochemistry

I confirm that I have read this submission and believe that I have an appropriate level of expertise to confirm that it is of an acceptable scientific standard.

# Peer review: Reviewer response for version 1

**DOI:** 10.5256/f1000research.30151.r75167

**Published:** 2020-12-16

**Authors:** Paul Skipp

**Affiliations:** 1 Centre for Proteomics Research, University of Southampton, Southampton, UK

The authors present valuable preliminary data investigating inflammatory signatures in patients previously infected with SARS-CoV2, 40 days after a positive diagnosis. Using a comprehensive mass spectrometry based multiplexed panel of 96 proteins associated with immune response, the study provides clear evidence of perturbed biochemical and inflammatory signature(s) 40 days after infection, even in asymptomatic patients. This provides important findings and a very useful methodology for future follow-up studies in Long COVID-19 cohorts.

 Minor suggestion for improvement of the manuscript:

- Please provide a reference(s) supporting the statement,"Work has been performed to characterise the inflammatory response to SARS-CoV2 infection in relation to disease severity".
- Ultimately, the mass spectrometry based MRM assay used in this study also only measures a defined panel of targets and hence pathways. In this light, I would suggest expanding or amending the statement, "There are sets of simple but expensive immunoassay panels that are available to look at known key inflammatory proteins such as cytokine panels; however, these only give information on known pathways and limit discovery of novel or less defined inflammatory responses".
- It would be useful to have a couple of sentences to give the reader an overview of the pathways that the 96 protein panel targets (i.e. NF-kB, MAPK, JAK-STAT, etc).
- The study provides a very useful and valuable methodology and although documented on protocols.io, a more comprehensive description in the main body of the manuscript would be useful.
- Please clarify Figure 1. Figure 1C does not exist. Was this figure a loading plot to show how strongly each characteristic influences a principal component?
- In the manuscript the authors state the following: "Figure 2 shows the univariate analysis of six proteins from our panel that were significantly altered". Please can you clarify in the text whether these were the only six proteins that were significantly altered or a selected subset.

Is the work clearly and accurately presented and does it cite the current literature?

Partly

If applicable, is the statistical analysis and its interpretation appropriate?

Yes

Are all the source data underlying the results available to ensure full reproducibility?

Yes

Is the study design appropriate and is the work technically sound?

Yes

Are the conclusions drawn adequately supported by the results?

Yes

Are sufficient details of methods and analysis provided to allow replication by others?

Yes

Reviewer Expertise:

Precision Medicine, Proteomics, Clinical Proteomics, Data Science, Biochemistry

I confirm that I have read this submission and believe that I have an appropriate level of expertise to confirm that it is of an acceptable scientific standard.

# Response

**Published:** 2020-12-18

**Authors:** Wendy Heywood

**Affiliations:** UCL, UK

Many thanks for taking the time to review our brief report. We have made all the suggested corrections including rewording and expanding of some comments as well as added additional reference. We have tried to improve the method description but the journal requirements require the methods section to be as brief as possible.

# Peer review: Reviewer response for version 1

**DOI:** 10.5256/f1000research.30151.r75166

**Published:** 2020-12-07

**Authors:** William J. griffiths

**Affiliations:** 1 Institute of Life Science, Swansea University Medical School, Swansea, UK; 2 Swansea University, Swansea, UK

This is an interesting study looking at a panel of inflammation-related protein signatures in a small cohort of patients 40+ days post infection. The authors see some changes in inflammatory proteins even this far after infection.

 The methods and underlying data are available at open access repositories.

 I just have a few minor suggestions:

1. Analysis section - It would be good to read a few lines on how the data was normalised (i.e. normalisation of protein concentration).
2. Results - I think the authors mean Figure 1a and 1b?

In summary, a nice brief study with some interesting results.

Is the work clearly and accurately presented and does it cite the current literature?

Yes

If applicable, is the statistical analysis and its interpretation appropriate?

Yes

Are all the source data underlying the results available to ensure full reproducibility?

Yes

Is the study design appropriate and is the work technically sound?

Yes

Are the conclusions drawn adequately supported by the results?

Yes

Are sufficient details of methods and analysis provided to allow replication by others?

Partly

Reviewer Expertise:

Mass spectrometry

I confirm that I have read this submission and believe that I have an appropriate level of expertise to confirm that it is of an acceptable scientific standard.

# Response

**Published:** 2020-12-18

**Authors:** Wendy Heywood

**Affiliations:** UCL, UK

Many thanks for reviewing our brief report. Additional information about how proteins were normalised has been included and the figure reference has been corrected in the results.
